# Determination of an optimal organ set to implement deformations to support four‐dimensional dose calculations in radiation therapy planning

**DOI:** 10.1120/jacmp.v9i2.2794

**Published:** 2008-04-28

**Authors:** Wafa Soofi, George Starkschall, Keith Britton, Sastry Vedam

**Affiliations:** ^1^ Department of Bioengineering Rice University Houston; ^2^ Departments of Radiation Physics The University of Texas M.D. Anderson Cancer Center Houston Texas; ^3^ Departments of Radiation Oncology The University of Texas M.D. Anderson Cancer Center Houston Texas

**Keywords:** 4D treatment planning, deformable image registration, respiratory motion

## Abstract

Surface‐based deformable image registration to generate a four‐dimensional (4D) dose calculation in radiation treatment planning requires the selection of a set of organ contours representing a basis set from which to generate anatomic deformation. The purpose of the present work was to determine the optimal set of organs needed to generate a basis set for deformation in treatment planning for thoracic tumors, such that the required computations are minimized, but that dose accuracy is high. Using retrospectively reviewed records and a deformable model algorithm in a research version of a commercial radiation treatment planning system, we calculated 4D dose distributions based on treatment plans for 10 patients with thoracic tumors. Various combinations of organs (total lungs, heart, spinal cord, external body surface) were used to generate the basis set used in the calculations for deformations. The external surface contour did not have a noticeable effect on the dose calculation. Total lung, heart, and spinal cord together provided an adequate set of deformation organs to generate accurate dose deformations. The magnitude of the calculated dose differences had no obvious relationship to tumor parameters, including site, histologic type, disease stage, extent of motion, or degree of centralization.

PACS numbers: 87.55.D‐, 87.55.dk, 87.55.kh

## I. INTRODUCTION

The anatomic deformation caused by respiratory activity constitutes an obstacle to the accuracy of dose calculations for radiation therapy of tumors located in the thorax. By explicitly imaging tumor movement, four‐dimensional (4D) computed tomography (CT), which involves the generation of sequential three‐dimensional (3D) image data sets representing phases of the patient's respiratory cycle, has provided greater accuracy in the radiation treatment of thoracic tumors.^(1)^ However, techniques that would account for motion‐induced deformation of tumor and surrounding tissues have not been well studied or clarified. Accurate calculation of the radiation dose distributions in a 4D CT data set requires a determination of the manner in which the 3D CT image of a reference phase deforms to all other phases in the 4D data set.

One technique for determining the deformation of a 3D CT data set is to track the deformation of specified anatomic structures from phase to phase in the 4D data set.^(^ ^2^ ^,^ ^3^ ^)^ In this approach, a tetrahedral mesh representing the surface of an anatomic structure is generated from contours drawn in the radiation treatment plan, and basis functions are used to interpolate deformations from the surface components through the volume of the known structure. This technique was recently incorporated into a research version of a commercial radiation treatment planning system (Pinnacle^3^, v8.1t: Philips Medical Systems, Milpitas, CA).^(4)^

The current implementation of 4D dose calculation requires that the user identify a set of organs that will undergo deformation. Points other than those explicitly identified in the mesh are interpolated using an elastic body spline deformation model, which characterizes the deformation of elastic bodies under the influence of elastically applied forces.^(4)^ The treatment planning system gives the user some flexibility in the selection of the organ surfaces that will represent the deformation of the thoracic cavity. In making that selection, the user must strike a balance between accuracy and efficiency. That is, the greater the number of organ surfaces selected, the more accurate the dose calculation is likely to be; however, because the chosen organs must be delineated onto the 3D data sets and mathematically deformed to simulate phases of respiration (a time‐consuming process), the minimum number of organ contours necessary for dose accuracy should be used in the calculations. The full set of deforming anatomic structures that can be used for this purpose consists of the lungs, heart, spinal cord, and external body surface. However, it may be that a subset of these organ contours can be utilized in dose calculations without unduly compromising dose accuracy. In the current study, we examined that possibility and also looked for correlations between tumor characteristics (size, histologic type and disease stage, degree of centralization within thoracic cavity, and extent of motion) and sensitivity to the organ contours used to approximate the thoracic deformation.

## II. MATERIALS AND METHODS

### A. Patient selection

In this institutional review board–approved study, we retrospectively reviewed the records of 10 patients who had undergone radiation treatment for tumors in the lung (n=8) or esophagus (n=2) and for whom 4D CT data sets had been acquired. Patient treatment plans were selected sequentially from the patient database in the treatment planning system and covered the period June and July 2006. Among the lung tumor patients, 1 received hypofractionated radiation treatments; the remaining 7 lung tumor patients and both patients with esophageal cancers received radiation using conventional fractionation schemes. Table 1 summarizes tumor site, disease stage, and prescription dose for the 10 patients.

**Table 1:** Patient number, tumor site, disease stage, and prescription dose

*Patient*	*Tumor histology and site*	*Disease stage*	*Prescription dose and fractionation scheme*
1	NSCLC left lung	T3N2M1	60 Gy, 20 fractions
2	NSCLC RLL (post surgery)	T2M2N0	50.4 Gy, 28 fractions
3	Adenocarcinoma distal esophagus	T3N1M0	50.4 Gy, 28 fractions
4	NSCLC RUL	T1N0M0	70 Gy, 35 fractions
5	NSCLC LUL	T1N2M0	63 Gy, 35 fractions
6	NSCLC LUL	T1N0M0	50 Gy, 4 fractions
7	Squamous cell carcinoma RLL	T2N2M0	63 Gy, 35 fractions
8	Adenocarcinoma RUL	T1N1M0, recurrent	63 Gy, 30 fractions
9	NSCLC RUL	T1N2M1	60 Gy, 30 fractions
10	Squamous mid esophagus	T3N1M0	50.4 Gy, 28 fractions

NSCLC=non‐small‐cell lung cancer;; RLL=right lower lobe;; RUL=right upper lobe;; LUL=left upper lobe.

### B. Treatment plans

Patients were immobilized using a conventional vacuum‐bag immobilization technique and were scanned using a multislice helical CT scanner (Discovery ST: GE Healthcare Systems, Waukesha, WI) operating in cine mode. The 4D CT image data sets, consisting of 3D CT data sets for each of 10 equally‐spaced phases of the respiratory cycle, were obtained using an image‐binning approach.^(1)^ Respiration was monitored using an external marker resting on the patient's abdomen (RPM: Varian Medical Systems, Palo Alto, CA). The CT images were reconstructed with a slice thickness of 2.5 mm.

We planned the treatments using the treatment planning system mentioned earlier, determining an explicit internal target volume (ITV) by mapping the motion of the clinical target volume (CTV) on the 4D CT data set. Tumor targeting was based on information in the 4D CT data set and was transferred to either a free‐breathing CT data set or an average CT data set, on which the treatment plan was developed. (An “average data set” is defined as a 3D CT data set in which each voxel value is the average value among the 10 phases that make up the 4D CT data set.)

In generating the planning target volume (PTV) for conventional fractionation plans, we established an isotropic 1.0‐cm margin around the ITV to account for setup uncertainty and interfractional variability in respiration. Conventionally fractionated patients were typically planned using inverse planning for intensity‐modulated radiation delivery with 5−7 6‐MV photon beams. For image‐guided hypofractionated treatments, the margin used to generate the PTV was 0.3 cm around the ITV. Patient immobilization was achieved with an extended vacuum bag, and image guidance was provided by daily in‐room CT imaging immediately before irradiation. Planning was accomplished using fixed 6‐MV photon beams. Anatomic structures that were normally contoured included the spinal cord, the right and left lungs, total lung, esophagus, and heart. Beam configurations were determined in a variety of ways, including conventional 3D conformal radiation therapy, intensity‐modulated radiation therapy, and image‐guided hypofractionated radiation therapy.

The treatment plans—including all CT data sets (planning set plus 10 phases comprising the 4D data set), the contours of the PTV and normal anatomic structures, and the beam configurations—were copied into a research directory, and the patients' identities were masked before further analysis was done.

### C. 4D dose calculations

To mathematically represent the deformation of the thoracic cavity throughout the respiratory cycle, we used the surface‐based deformable image registration method.^(^ ^2^ ^,^ ^3^ ^)^ Regions of interest (ROIs), which may be organs or target volumes, were delineated on a single reference 3D CT image data set either by the attending radiation oncologist or by the treatment planner. (In the latter case, plans were subsequently reviewed by the radiation oncologist.) A deformable model algorithm was then used to propagate the ROIs to the phases constituting the 4D CT image data set.^(^ ^5^ ^,^ ^6^ ^)^ The 4D dose distributions were calculated first by using the collapsed cone convolution algorithm to perform a dose calculation on each of the 10 phases of the 4D CT data set.^(7)^ One data set, typically the 0% phase data set, was identified as the reference data set, and each of the other data sets was deformed to the reference data set using the deformable image registration technique^(4)^ based on a specified set of ROIs and the elastic body spline deformation model. The dose matrix in each data set was then deformed to the reference data set based on the foregoing deformations, and the doses were calculated and accumulated.

Initially, the ROIs used for the deformation included the total lung (L), heart (H), spinal cord (C), and external body surface (E). This LHCE set of ROIs represented the highest attainable dose accuracy for the 4D dose calculation, because it included all anatomic structures that are routinely delineated in the thoracic region for treatment planning. We then recalculated the deformation using subsets of the control organ set by excluding selected contours from the deformation. The following sets of ROIs were used in the analysis: LHCE (control), LHC, LH, LC, LE, and L. In all cases, the total lung was included because it represents the major contribution to deformation.

### D. Dose–volume histogram analysis

First, we used the LHCE set of ROIs to generate control dose–volume histograms (DVHs) for each patient. Then, we used each of the subsets of ROIs to generate experimental DVHs based on 4D dose calculations. Finally, we compared the experimental DVHs with the control DVHs. We generated DVHs for the spinal cord, CTV, PTV, heart, right lung, left lung, and both lungs. Several parameters were selected to analyze the DVHs, including the V20 value (fractional volume of the ROI receiving at least 20 Gy) for the total lung ROI, the D5 value (dose received by 5% of the ROI volume) for the spinal cord, the D95 value (dose received by 95% of the ROI volume) for the PTV, and the V(prescription) value (fractional volume of the ROI receiving the prescription dose) for the CTV.

Total lung V20 was selected because it appears to be closely related to the risk of radiation pneumonitis.^(8)^ Although the maximum dose to the spinal cord is used to predict the risk of radiation myelitis,^(9)^ we studied the cord D5 value instead because the maximum dose to the cord may vary greatly for situations in which the DVH exhibits a long “tail.” (The D5 value is likely to be more consistent among deformation organ sets.) We selected D95 for the PTV because our clinical standard of practice is that 95% of the PTV receives the prescription dose. Finally, we selected V(prescription) for the CTV because our clinical standard of practice is that the entire CTV receives the prescription dose [that is, that V(prescription)=100%].

Deviations of the experimental DVHs from the control DVHs were visually characterized by means of composite DVHs and were then quantified using the differences between the D95 values of each organ subset and the control (LHCE) organ set. We organized the results by extent of deviation from control and then looked for trends that might be related to tumor size, histologic type, or disease stage; approximate degree of centralization; and extent of respiratory motion, which was approximated by measuring the range of motion of the apex of the right side of the diaphragm.

## III. RESULTS

**Figure 1:**
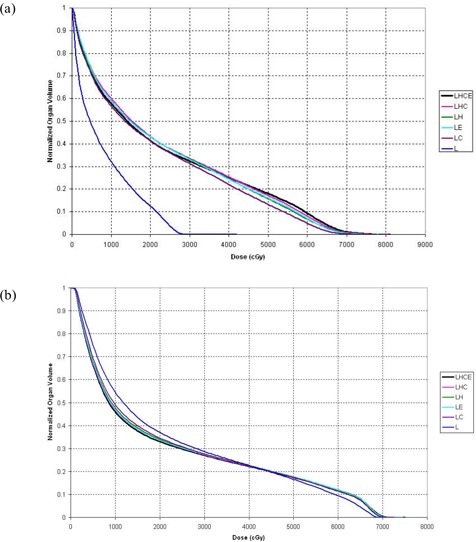
Cumulative dose–volume histograms (DVHs) for total lung. (a) The DVHs for patient 5 show large discrepancies between the deformation organ sets. (b) The DVHs for patient 7 are more typical of the discrepancies seen between organ sets for the entire group of patients. LHCE=four‐dimensional (4D) dose calculation driven by deformations of lung, heart, cord, external contour; LHC=4D dose calculation driven by deformations of lung, heart, cord; LH=4D dose calculation driven by deformations of lung, heart; LE=4D dose calculation driven by deformations of lung, external contour; LC=4D dose calculation driven by deformations of lung, cord; L=4D dose calculation driven by deformations of lung.

As a general rule, the DVHs for total lungs were similar, regardless of the organs used in the deformation. Fig. 1(a) shows the DVHs for total lung for patient 5, who showed the largest difference in DVHs calculated using various organ sets; Fig. 1(b) shows the DVHs for total lung for patient 7, whose results were more typical of the entire group of 10 patients. Both figures demonstrate that the DVHs for each ROI set were quite similar, with the exception of the DVH based solely on deformation of the lung contours. Table 2 summarizes the V20 values, showing deformations based on each combination of ROIs. These data indicate that the LHC deformation organ set yielded V20 values that most closely approximated those obtained with the full LHCE set [root mean square (RMS) deviation: 1.25%; maximum deviation: 2.59%], although the LH and LE deformation sets yielded comparable values. In general, deformation based on L alone gave the poorest agreement with LHCE, although the LC sometimes yielded even more divergent results.

Table 3 shows the data from Table 2 rearranged in descending order of absolute difference between the LHC‐ and LHCE‐generated total lung V20 values. Tumor site, disease stage, prescription dose, and hypofractionation status are also indicated. Visual inspection of the data detected no trends linking the differences in V20 values with tumor site, disease stage, or fractionation scheme.

**Table 2:** Values of V20 for total lung, and differences between control and experimental values for each of the 10 study patients, using varying combinations of regions of interest to drive the deformations in the four‐dimensional dose calculations

*Patient*	*V20 values (%)*						*V20 differences (%)*				
	*LHCE*	*LHC*	*LH*	*LE*	*LC*	*L*	LHC−LHCE LHC−LHCE	LH−LHCE LH−LHCE	LE−LHCE	LC−LHCE	L−LHCE
1	26.26	26.34	26.37	26.46	26.58	26.62	0.08	0.11	0.20	0.32	0.36
2	21.52	22.21	22.93	22.74	23.72	27.09	0.69	1.41	1.22	2.20	5.57
3	19.93	21.11	21.06	19.12	20.70	17.98	1.19	1.13	−0.80	0.77	−1.94
4	28.91	28.88	28.86	28.90	28.85	28.77	−0.03	−0.05	−0.01	−0.07	−0.14
5	41.40	43.22	43.04	43.05	41.40	12.47	1.82	1.64	1.64	−0.01	−28.94
6	2.92	2.64	2.38	2.02	1.11	0.51	−0.29	−0.55	−0.91	−1.82	−2.42
7	32.99	33.50	34.00	33.53	34.56	36.98	0.52	1.01	0.54	1.57	3.99
8	38.10	39.24	39.72	41.28	44.73	41.96	1.14	1.63	3.18	6.63	3.86
9	36.51	37.98	37.86	36.60	38.91	38.65	1.47	1.35	0.08	2.40	2.14
10	27.89	30.48	31.35	29.95	31.89	31.76	2.59	3.46	2.06	4.00	3.87
RMS deviation							1.25	1.54	1.43	2.78	9.64
Maximum deviation							2.59	3.46	3.18	6.63	28.94

V20=fractional volume of anatomic structure receiving at least 20 Gy; LHCE=4D dose calculation driven by deformations of lung, heart, cord, external contour; LHC=4D dose calculation driven by deformations of lung, heart, cord; LH=4D dose calculation driven by deformations of lung, heart; LE=4D dose calculation driven by deformations of lung, external contour; LC=4D dose calculation driven by deformations of lung, cord; L=4D dose calculation driven by deformations of lung; RMS=root mean square; root mean square.

**Table 3:** Patients sorted in descending order by absolute value of the difference between total lung V20 obtained by using lung, heart, cord (LHC) for deformation and total lung V20 obtained by using lung, heart, cord, external contour (LHCE) for lung deformation

*Patient*	*Total lung V20 difference (%)*	*Tumor site*	*Disease stage*	*Prescription dose and fractionation scheme*	*Hypofx?*
10	2.59	Squamous mid‐esophagus	T3N1M0	50.4 Gy, 28 fractions	No
5	1.82	NSCLC LUL	T1N2M0	63 Gy, 35 fractions	No
9	1.47	NSCLC RUL	T1N2M1	60 Gy, 30 fractions	No
3	1.19	Adenocarcinoma, distal esophagus	T3N1M0	50.4 Gy, 28 fractions	No
8	1.14	Adenocarcinoma RUL	T1N1M0 recurrent	63 Gy, 30 fractions	No
2	0.69	NSCLC RLL (post surgery)	T2M2N0	50.4 Gy, 28 fractions	No
7	0.52	Squamous‐cell carcinoma RLL	T2N2M0	63 Gy, 35 fractions	No
6	−0.29	NSCLC LUL	T1N0M0	50 Gy, 4 fractions	Yes
1	0.08	NSCLC left lung	T3N2M1	60 Gy, 20 fractions	No
4	−0.03	NSCLC RUL	T1N0M0	70 Gy, 35 fractions	No

V20=fractional volume of anatomic structure receiving at least 20 Gy; Hypofx=hypofractionated; NSCLC=non‐small‐cell lung cancer; LUL=left upper lobe; RUL=right upper lobe; RLL=right lower lobe.

**Figure 2:**
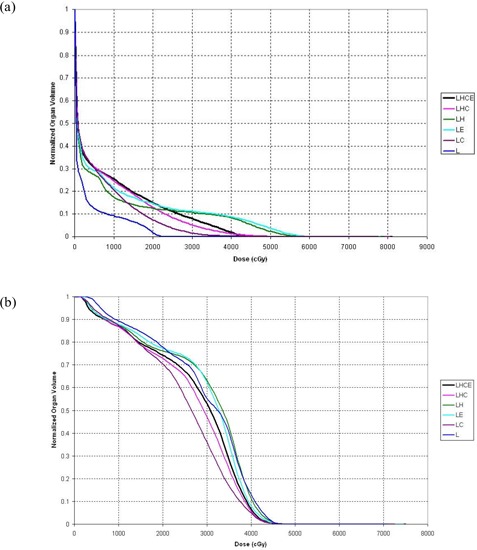
Cumulative dose–volume histograms (DVHs) for spinal cord. (a) The DVHs for patient 5 show large discrepancies between the deformation organ sets. (b) The DVHs for patient 7 are more typical of the discrepancies seen between organ sets for the entire group of patients. LHCE=four‐dimensional (4D) dose calculation driven by deformations of lung, heart, cord, external contour; LHC=4D dose calculation driven by deformations of lung, heart, cord; LH=4D dose calculation driven by deformations of lung, heart; LE=4D dose calculation driven by deformations of lung, external contour; LC=4D dose calculation driven by deformations of lung, cord; L=4D dose calculation driven by deformations of lung.

Table 4 shows values of D5 and differences between D5 values calculated using each subset of deformation organs and the control set. Again, calculations based on the LHC subset had the smallest RMS deviation (235 cGy) and the smallest maximum deviation (500 cGy). Fig. 2(a) shows the cord DVHs for patient 5, for whom the disagreement between calculations was large, and Fig. 2(b) shows the cord DVHs for patient 7, whose results were more typical of the entire patient group. The cord dose calculated for patient 5 using the LHC deformation organ set would most likely be viewed as clinically acceptable, because a negligible amount of cord is calculated to receive a dose greater than 45 Gy, but the cord doses calculated using the LH and LE data sets likely would not be considered clinically acceptable because a non‐negligible amount of cord is calculated to receive a dose greater than 50 Gy.

Table 5 shows the data in Table 4 rearranged in descending order of the absolute difference between the LHC‐ and LHCE‐generated cord D5 value, together with tumor site, disease stage, prescription dose, and hypofractionation status. Although the absolute D5 differences for esophagus treatments are clustered, the sample is too small for that clustering to be considered any sort of a trend. The difference in D5 values for the hypofractionated treatment was 0, but that result may simply have been the result of the spinal cord receiving very little dose in the hypofractionated treatment.

Table 6 compares values of D95 for the PTV, together with the differences in D95 between the control calculations and those derived using each subset of deformation organs. Once again, the LHC subset exhibited the smallest RMS deviation (232 cGy) and the smallest maximum deviation (500 cGy). For many patients, the differences in the dose calculated using only the lungs as the deformation organ set and the dose calculated using the full LHCE organ set were so great that the L subset dose distribution would be viewed as clinically unacceptable.

**Figure 3:**
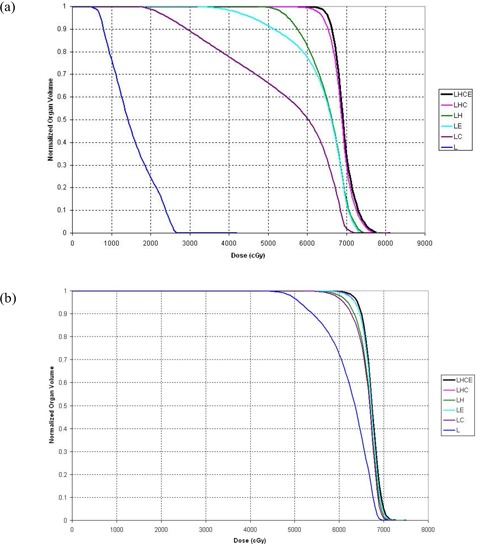
Cumulative dose–volume histograms (DVHs) for the clinical target volume. (a) The DVHs for patient 5 show large discrepancies between the deformation organ sets. (b) The DVHs for patient 7 are more typical of the discrepancies between organ sets for the entire group of patients. LHCE=four−dimensional (4D) dose calculation driven by deformations of lung, heart, cord, external contour; LHC=4D dose calculation driven by deformations of lung, heart, cord; LH=4D dose calculation driven by deformations of lung, heart; LE=4D dose calculation driven by deformations of lung, external contour; LC=4D dose calculation driven by deformations of lung, cord; L=4D dose calculation driven by deformations of lung.

Finally, Table 7 shows the values of V(prescription) for the CTV and the differences in V(prescription) between calculations performed using each subset of deformation organs and those performed using the LHCE set of organs. In some cases, the volume receiving the actual prescription dose was somewhat smaller than originally intended, and thus the volume receiving the lower dose is recorded here. Again, the LHC subset exhibited the smallest RMS deviation (4.59%) and the smallest maximum deviation (11.88%). In several cases, use of the total lung as the only ROI for deformation resulted in severe underdosing of the CTV. Figs. 3(a) and 3(b), for patients 5 and 7 respectively, vividly illustrate that result. The data sets for patient 5, which exhibited the largest disagreement between other organs, also had a large (but not the largest) disagreement for the CTV; the degree of the disagreements between the data sets for patient 7 were more typical of those for the whole group. For patient 5, the only organ subset that gave a DVH for the CTV comparable to that calculated using the LHCE organ set was the LHC set; DVHs calculated using the other sets suggested that the doses were clinically unacceptable. For patient 7, DVHs calculated using the LHC and LE organ sets were comparable to those from the LHCE calculations, and DVHs derived using LC and LH indicated doses that likely would be considered clinically acceptable; however, DVHs calculated using L alone were clinically unacceptable.

**Table 4:** Values of D5 for spinal cord and differences in D5 values relative to control for each of the 10 study patients, using varying combinations of regions of interest to drive the deformations in the four‐dimensional (4D) dose calculations

*Patient*	*D5 values (cGy)*						*D5 differences (cGy)*				
	*LHCE*	*LHC*	*LH*	*LE*	*LC*	*L*	LHC−LHCE LHC−LHCE	LH−LHCE LH−LHCE	LE−LHCE	LC−LHCE	L−LHCE
1	3550	3550	3550	3550	3550	3550	0	0	0	0	0
2	3750	3700	4000	3800	3600	2750	−50	250	50	−150	−1000
3	3900	3750	3550	3800	3150	3250	−150	−350	−100	−750	−650
4	1650	1700	1900	1800	1650	1850	50	250	150	0	200
5	3500	3000	4550	4750	2300	1700	−500	1050	1250	−1200	−1800
6	400	400	450	450	350	400	0	50	50	−50	0
7	4050	4050	4200	4100	4000	4250	0	150	50	−50	200
8	4500	4500	4650	4700	4200	4300	0	150	200	−300	−200
9	4100	4600	4300	4050	4650	4200	500	200	−50	550	100
10	4650	4800	5150	5100	4650	4300	150	500	450	0	−350
RMS deviation							235	411	430	492	701
Maximum deviation							500	1050	1250	1200	1800

D5=maximum dose received by at least 5 % of spinal cord; LHCE=4D dose calculation driven by deformations of lung, heart, cord, external contour; LHC=4D dose calculation driven by deformations of lung, heart, cord; LH=4D dose calculation driven by deformations of lung, heart; LE=4D dose calculation driven by deformations of lung, external contour; LC=4D dose calculation driven by deformations of lung, cord; L=4D dose calculation driven by deformations of lung; RMS=root mean square.

**Table 5:** Patients sorted in descending order by absolute value of the difference between cord D5 obtained by using lung, heart, cord (LHC) for deformation and total lung D5 value obtained by using lung, heart, cord, external contour (LHCE) for lung deformation

*Patient*	*Cord D5 difference (cGy)*	*Tumor site*	*Disease stage*	*Prescription dose and fractionation scheme*	*Hypofx?*
5	−500	NSCLC LUL	T1N2M0	63 Gy, 35 fractions	No
9	500	NSCLC RUL	T1N2M1	60 Gy, 30 fractions	No
3	−150	Adenocarcinoma, distal esophagus	T3N1M0	50.4 Gy, 28 fractions	No
10	150	Squamous mid‐esophagus	T3N1M0	50.4 Gy, 28 fractions	No
2	−50	NSCLC RLL (post surgery)	T2M2N0	50.4 Gy, 28 fractions	No
4	50	NSCLC RUL	T1N0M0	70 Gy, 35 fractions	No
8	0	Adenocarcinoma RUL	T1N1M0 recurrent	63 Gy, 30 fractions	No
7	0	Squamous cell carcinoma RLL	T2N2M0	63 Gy, 35 fractions	No
6	0	NSCLC LUL	T1N0M0	50 Gy, 4 fractions	Yes
1	0	NSCLC left lung	T3N2M1	60 Gy, 20 fractions	No

D5=maximum dose received by at least 5% of spinal cord; Hypofx=hypofractionated; NSCLC=non‐small‐cell lung cancer; LUL=left upper lobe; RUL=right upper lobe; RLL=right lower lobe.

**Table 6:** Values of D95 for the planning target volume and differences in D95 values relative to control for each of the 10 study patients, using varying combinations of regions of interest to drive the deformations in the four‐dimensional (4D) dose calculations

*Patient*	*D95 values (cGy)*						*D95 differences (cGy)*				
	*LHCE*	*LHC*	*LH*	*LE*	*LC*	*L*	LHC−LHCE LHC−LHCE	LH−LHCE LH−LHCE	LE−LHCE	LC−LHCE	L−LHCE
1	5500	5500	5500	5550	5550	5550	0	0	50	50	50
2	4750	4850	4450	4550	4500	2950	100	−300	−200	−250	−1800
3	4650	4600	4050	4350	3450	900	−50	−600	−300	−1200	−3750
4	7100	7050	6950	6950	6600	6550	−50	−150	−150	−500	−550
5	5100	4850	3750	2600	1450	500	−250	−1350	−2500	−3650	−4600
6	3900	3400	2950	2400	1450	1100	−500	−950	−1500	−2450	−2800
7	5950	5850	5650	5800	5500	4650	−100	−300	−150	−450	−1300
8	5650	5450	5200	5000	4750	3350	−200	−450	−650	−900	−2300
9	4500	4500	4500	4350	4050	4150	0	0	−150	−450	−350
10	4550	4150	2650	2800	2200	500	−400	−1900	−1750	−2350	−4050
RMS deviation							232	843	1104	1668	2648
Maximum deviation							500	1900	2500	3650	4600

D95=maximum dose received by at least  5% of planning target volume;LHCE=4D dose calculation driven by deformations of lung, heart, cord, external contour; LHCE=4D dose calculation driven by deformations of lung, heart, cord;LH=4D dose calculation driven by deformations of lung, heart;LE=4D dose calculation driven by deformations of lung, external contour; LC=4D dose calculation driven by deformations of lung, cord; L=4D dose calculation driven by deformations of lung; RMS = root mean square.

**Table 7:** Values of V(prescription) for the planning target volume and differences in V(prescription) values relative to control for each of the 10 study patients,^a^ using varying combinations of regions of interest to drive the deformations in the four‐dimensional (4D) dose calculations

*Patient*	*Prescription dose (Gy)*	*V(prescription) values (%)*						*V(prescription) differences (%)*				
		*LHCE*	*LHC*	*LH*	*LE*	*LC*	*L*	*LHC– LHCE*	*LH– LE– LHCE LHCE*	*LC– LHCE*	*L– LHCE*
1	54.0^b^	99.89	99.86	99.86	99.97	99.96	99.96	0.00	−0.03	−0.03	0.08	0.07
2	50.4	99.11	99.71	99.15	99.81	99.99	48.31	0.60	0.04 0.70	0.88	−50.80
3	45.0^c^	99.00	98.99	96.14	99.11	94.04	65.67	−0.01	−2.86	0.11	−4.96	−33.33
4	70.0	100.00	100.00	99.67	99.84	89.33	74.35	0.00	−0.33	−0.16	−10.67	−25.65
5	63.0	99.36	97.13	69.93	67.83	41.75	0.00	−2.23	−29.43	−31.53	−57.61	−99.36
6	40.0^d^	99.63	93.51	80.33	60.79	13.36	0.00	−6.12	−19.30	−38.84	−86.27	−99.63
7	63.0	97.86	96.54	91.53	96.26	88.77	54.13	−1.32	−6.33	−1.60	−9.09	−43.73
8	63.0	77.10	73.32	65.51	61.34	45.36	12.23	−3.79	−11.60	−15.76	−31.74	−64.87
9	60.0	79.32	77.91	74.69	75.76	51.85	41.31	−1.41	−4.63	−3.57	−27.47	−38.02
10	50.4	96.72	89.97	69.03	75.55	67.28	18.12	−6.75	−27.69	−21.16	−29.43	−78.60
RMS deviation								3.26	14.87	17.93	36.89	61.56
Maximum deviation								6.75	29.43	38.84	86.27	99.63

a In 3 cases in which the plan actually delivered a lower dose to the clinical target volume (CTV), the actual dose delivered to the CTV is identified as the prescription dose.

b The prescription dose of 60.0 Gy was to be delivered to the gross tumor volume. The prescription to the CTV was 54.0 Gy.

c Although the prescription dose was 50.4 Gy, the plan actually delivered 45.0 Gy to the CTV.

d Although the prescription dose was 50.0 Gy, the plan actually delivered 40.0 Gy to the CTV.

V(prescription) = fractional volume of planning target volume receiving at least prescription dose; LHCE=4D dose calculation driven by deformations of lung, heart, cord, external contour; LHC=4D dose calculation driven by deformations of lung, heart, cord; LH=4D dose calculation driven by deformations of lung, heart; LE=4D dose calculation driven by deformations of lung, external contour; LC=4D dose calculation driven by deformations of lung, cord; L=4D dose calculation driven by deformations of lung; RMS = root mean square.

Table 8 shows the data from Table 7 rearranged in descending order of absolute difference between the LHC‐ and LHCE‐generated V(prescription) values for the CTV. Tumor site, disease stage, prescription dose, and hypofractionation status are also indicated. Visual inspection of this table suggested no trend linking the differences in V(prescription) values with tumor site, disease stage, or fractionation status.

**Table 8:** Patients sorted in descending order by absolute value of the difference between V(prescription) for the clinical target volume (CTV) obtained by using lung, heart, cord (LHC) for deformation and V(prescription) for the CTV obtained by using lung, heart, cord, external contour (LHCE) for lung deformation

*Patient*	*CTV V(prescription) difference*	*Tumor site*	*Disease stage*	*Prescription dose and fractionation scheme*	*Hypofx?*
10	−6.75%	Squamous mid‐esophagus	T3N1M0	50.4 Gy, 28 fractions	No
6	−6.12%	NSCLC LUL	T1N0M0	50 Gy, 4 fractions	Yes
8	−3.79%	Adenocarcinoma RUL	T1N1M0 recurrent	63 Gy, 30 fractions	No
5	−2.23%	NSCLC LUL	T1N2M0	63 Gy, 35 fractions	No
9	−1.41%	NSCLC RUL	T1N2M1	60 Gy, 30 fractions	No
7	−1.32%	Squamous cell carcinoma RLL	T2N2M0	63 Gy, 35 fractions	No
2	0.60%	NSCLC RLL (post surgery)	T2M2N0	50.4 Gy, 28 fractions	No
3	−0.01%	Adenocarcinoma, distal esophagus	T3N1M0	50.4 Gy, 28 fractions	No
1	0.00%	NSCLC left lung	T3N2M1	60 Gy, 20 fractions	No
4	0.00%	NSCLC RUL	T1N0M0	70 Gy, 35 fractions	No

V(prescription)=fractional volume of planning target volume receiving at least prescription dose; NSCLC=non‐small‐cell lung cancer; LUL=left upper lobe; RUL=right upper lobe; RLL=right lower lobe.

## IV. DISCUSSION

To calculate 4D dose distributions, a Cartesian dose calculation matrix is generated on each phase in the 4D CT data set, the dose distribution on each of the dose matrices is calculated, and then each dose matrix is deformed to the reference phase, accumulating the dose on the reference matrix for that phase. The difficult part is the deformation of each phase, because the matrices that define the deformation ideally indicate how each tissue volume element moves between phases during respiration.

Current approaches to deformable registration fall into two classes: image‐based deformations and model‐based deformations.

Image‐based deformations, as exemplified by algorithms such as the “demons” algorithm^(10)^ and optical flow methods,^(11)^ generate deformations based on the values of the voxels in the CT image data sets. Although user intervention may not be needed to effect those deformations, one‐to‐one correspondence between CT voxels and tissue volume elements is not guaranteed. In an extreme case, in which deformation of a homogeneous medium occurs, image‐based deformable registration methods may not be able to detect the magnitude or even the presence of a deformation.

Model‐based deformations, such as that described by Kaus et al.^(4)^ and implemented in the commercial radiation treatment planning system used here, require pre‐processing in the form of segmentation of each data set to generate the contours of corresponding ROIs. Additional information in the form of basis functions and biomechanical information^(12)^ may have to be introduced to interpolate deformations into the interior of segmented anatomic structures. In addition, some judgment is needed to select a set of organ contours sufficient to accurately reflect the totality of deformations that the interior of a patient undergoes during the respiratory cycle.

On the basis of our findings, we conclude that accurate doses can be calculated in 4D CT planning of radiation treatments using primarily internal organ contours for image deformation, rather than the full set of contours typically used, including those for external body surface. As seen in the present work, selecting simply the total lungs as the deformation organ does not adequately characterize the deformation needed to accurately calculate the 4D dose distribution. It appears that organs such as the heart and spinal cord, medial structures that do not deform as extensively as the lungs, are needed to calculate dose accurately. However, including more organs than is necessary slows down the deformation process in two ways. First, organ contours have to be generated on each phase of the 4D data set. That task is accomplished by copying the mesh model of the organ from the reference CT data set to each of the other phases, and then by deforming the model to fit the specific images on the phases. Second, the deformations of all organs have to be incorporated into the deformation calculations. Consequently, a good compromise needs to be found to limit the number of organs included in the deformations.

In the present work, we found little difference between DVHs generated using the original organ set (LHCE) and those generated using the experimental subset LHC, suggesting that the external body surface contour does not necessarily play a large role in characterizing data set deformations. Calculations based on deformations using other organ subsets tended to exhibit substantial variation, with the DVH generated using the lung contour alone generally being the least accurate.

Tables 3, 5, and 8 reveal no obvious trend linking the differences in the DVHs generated using the LHC organ subset and the full LHCE set with the tumor site or disease stage. Patient 6, who received hypofractionated radiation treatment, had a larger‐than‐typical discrepancy between CTV dose calculations; however, that patient had less discrepancy in dose calculations for other ROIs. For most other patients, there appeared to be correlation between DVH consistencies among ROIs.

Reducing the number of organs used for a deformation does not always result in a less accurate calculation. For instance, the doses calculated for Patient 1 appeared to depend solely on the deformation of the lungs. The reason for this complete insensitivity of dose to deformation of the heart, spinal cord, and external contours is unclear.

We further found in a partial study that inclusion of the GTV as a deforming organ does not appear to affect the dose calculation.

The substantial increase in the accuracy of predicted radiation doses to thoracic tumors afforded by 4D dose calculations may result in more effective treatment planning. As noted here, a major limiting factor in the practicality of the 4D approach, the volume of data calculations required, may be reduced if further studies can determine precisely which tumor parameters affect the sensitivity of the dose calculation to the organ subset. If tumor characteristics can be correlated with the organ subsets required for an accurate dose calculation, then it may be possible, through the visualization of CT scans alone, to individually tailor the organ sets used in each treatment plan to generate a dose calculation with an ideal balance between accuracy and efficiency.

Finally, it should be noted that the present study has demonstrated that the LHC subset of organs gives the same dose distributions as the complete LHCE set of organs, but does not make any statements regarding the absolute accuracy of 4D dose calculations. Comparison with measurements would verify the accuracy of 4D dose calculations, and such work is in progress.

## V. CONCLUSIONS

The present work has demonstrated that, for 4D dose calculations in the thorax using a surface‐based deformable image registration approach based on deforming contours of specific organs, a minimum set of total lung, heart, and spinal cord is necessary to give results comparable to calculations using all organs.

## ACKNOWLEDGMENTS

The present work was supported in part by a Sponsored Research Agreement from Philips Medical Systems. The authors also acknowledge the assistance of Dr. Veni Ezhil and Michael Kantor.

## Supporting information

Supplementary Material
