# Supplementary material for: Determination of an optimal organ set to implement deformations to support four‐dimensional dose calculations in radiation therapy planning
Source: J Appl Clin Med Phys. 2008 Apr 28;9(2):69–82. doi: 10.1120/jacmp.v9i2.2794 (PMC5721707; doi:10.1120/jacmp.v9i2.2794)
Supplement: Supplementary file 1 — Supplementary Material [file ACM2-9-069-s001.doc]

Determination of an optimal organ set to implement deformations to support four-dimensional dose calculations in radiation therapy planning

Wafa Soofi,^1,2^ George Starkschall, ^2^ Keith Britton, ^2,3^ and Sastry Vedam^2^

^1^Department of Bioengineering, Rice University, Houston, TX; Departments of ^2^Radiation Physics and ^3^Radiation Oncology, The University of Texas M. D. Anderson Cancer Center, Houston, TX

Corresponding author: George Starkschall, Ph.D.,

Department of Radiation Physics, Unit 94,

The University of Texas M. D. Anderson Cancer Center,

1515 Holcombe Blvd., Houston, TX 77030, U.S.A

Telephone: (713) 563-2537

Fax: (713) 563-2479

Email: [gstarksc@mdanderson.org](mailto:gstarksc@mdanderson.org)

Running title: Organ deformations in 4D dose calculations
